# Regulatory role of cytosolic phospholipase A_2_ alpha in the induction of CD40 in microglia

**DOI:** 10.1186/s12974-017-0811-z

**Published:** 2017-02-10

**Authors:** Yafa Fetfet Malada-Edelstein, Nurit Hadad, Rachel Levy

**Affiliations:** Infectious Diseases Laboratory, Department of Clinical Biochemistry and Pharmacology, Faculty of Health Sciences, Ben-Gurion University of the Negev, 84105 Beer-Sheva, Israel

**Keywords:** Cytosolic phospholipase A_2_α, CD40, Microglia, Lipopolysaccharide, Interferon gamma, Nuclear factor-κB

## Abstract

**Background:**

The aberrant expression of CD40, a co-stimulatory receptor found on the antigen-presenting cells, is involved in the pathogenesis of various degenerative diseases. Our previous study demonstrated that the reduction of cytosolic phospholipase A_2_ alpha (cPLA_2_α) protein overexpression and activation in the spinal cord of a mouse model of ALS, hmSOD1 G93A, inhibited CD40 upregulation in microglia. The present study was designed to determine whether cPLA_2_α has a direct, participatory role in the molecular events leading to CD40 induction.

**Methods:**

Cultures of primary mouse microglia or BV-2 microglia cell line exposed to lipopolysaccharide (LPS) or interferon gamma (IFNγ) for different periods of time, in order to study the role of cPLA_2_α in the events leading to CD40 protein induction.

**Results:**

Addition of LPS or IFNγ caused a significant upregulation of cPLA_2_α and of CD40, while prevention of cPLA_2_α upregulation by a specific oligonucleotide antisense (AS) prevented the induction of CD40, suggesting a role of cPLA_2_α in the induction of CD40. Addition of LPS to microglia caused an immediate activation of cPLA_2_α detected by its phosphorylated form, while addition of IFNγ induced cPLA_2_α activation at a later time scale (4 h). The activation of cPLA_2_α is mediated by ERK activity. Suppression of cPLA_2_α activity inhibited superoxide production by NOX2-NADPH oxidase and activation of NF-κB detected by the phosphorylation of p65 on serine 536 at 15 min by LPS and at 4 h by IFNγ. Inhibition of NOX2 prevented NF-κB activation and CD40 induction but did not affect cPLA_2_α activation, suggesting cPLA_2_α is located upstream to NOX2 and NF-κB. The activation of cPLA_2_ by LPS was mediated by both adaptor proteins downstream to LPS receptor; TRIF and MyD88, while the activation of cPLA_2_α by IFNγ was mediated by the secreted TNF-α at 4 h. The early activation of STAT1α (detected by phospho-serine727 and phoshpo-tyrosine701) by IFNγ and the late activation of STAT1α by LPS were not affected in the presence of cPLA_2_α inhibitors, indicating that STAT1α is not under cPLA_2_α regulation.

**Conclusions:**

Our results show for the first time that cPLA_2_ upregulates CD40 protein expression induced by either LPS or IFNγ, and this regulatory effect is mediated via the activation of NOX2-NADPH oxidase and NF-κB. Cumulatively, our results indicate that cPLA_2_α may serve as a pivotal amplifier of the inflammatory response in the CNS.

## Background

The co-stimulatory receptor, CD40 molecule, is a 50-kDa type I member of the tumor necrosis factor receptor superfamily that is widely expressed by the various immune and non-immune cells [1–7]. The interaction between CD40 and its ligand, CD40L (CD154), is one of multiple signals necessary for a productive immune response [8–10]. The CD40-CD154 interaction promotes a wide spectrum of molecular and cellular processes including, immunoglobulin class switching, cell differentiation and maturation, B-cell growth, and expression of other co-stimulatory molecules such as MHC class II, ICAM-1, VCAM-1, E-selectin, LFA-3, B7.1, and B7.2) [11, 12]. In addition, CD40-CD154 interaction induces the production of cytotoxic radicals and of various pro-inflammatory cytokines (TNF-α, IL6, IL-8, and IL-12) and chemokines (CCL-2) [13, 14].

In the central nervous system (CNS), the microglial cells are constantly in motion, surveying their environment to protect the nervous system acting as debris scavengers, killers of pathogens, and regulators of innate and adaptive immune responses. The microglia cells express the key surface molecules for antigen presentation (CD40, MHC-II, and B7); therefore, they are considered the most potent endogenous antigen-presenting cells in the CNS [15]. In a healthy nervous system, microglia constitutively expresses CD40 at a low level, which is enhanced under inflammatory conditions. Several studies show that the aberrant expression of CD40 is involved in the initiation and maintenance of various neurodegenerative diseases including multiple sclerosis, Alzheimer’s disease, HIV-1-associated dementia and cerebral ischemia [16–20], and other diseases as rheumatoid arthritis and atherosclerosis [18, 21, 22]. Blockade of CD40-CD40L signaling has been shown to provide a significant beneficial effect in a number of animal models of neurological human diseases [1, 18, 23–28].

Previous findings suggested that cPLA_2_α plays an important role in inflammation. cPLA_2_α specifically hydrolyzes phospholipids containing arachidonic acid at the sn-2 position [29, 30] and is generally thought to be the rate-limiting step in the generation of eicosanoids and platelet activating factor. These lipid mediators play critical roles in the initiation and modulation of inflammation and oxidative stress. cPLA_2_α is ubiquitous in the brain cells and is essential for their physiological regulation. However, elevated cPLA_2_α expression and activity were detected in the inflammatory sites in a vast array of inflammatory diseases [31], including neurodegenerative diseases such as Alzheimer’s disease, multiple sclerosis, and amyotrophic lateral sclerosis (ALS) [32–35]. Our previous study [36] in a mouse model of ALS, hmSOD1 G93A, demonstrated that the blunting cPLA_2_α protein expression and inhibition of its activity inhibited microglial-CD40 upregulation. This inhibitory effect could be a result of a direct regulatory role of cPLA_2_α on CD40 inductive process or an indirect effect due to damping of inflammation. The present study was designed to determine whether cPLA_2_α has a direct role in the events leading to CD40 protein induction. To this aim, we used mouse microglia cultures and two different stimuli, LPS and IFNγ that have been reported to induce CD40 upregulation. The signal transduction events leading to CD40 upregulation by both stimuli have been studied, and it was reported that they include two transcription factors NF-κB and STAT1α that are activated in different rank order and time scale by the two stimuli [37–39].

## Methods

### Materials

Glutamine, penicillin-streptomycin-nystatin, phosphate buffered saline (PBS) Dulbecco’s Modified Eagle’s Medium (DMEM), Hanks’ Balanced Salts Solution (HBSS), fetal bovine serum (FBS), HEPES, sodium pyruvate, Dulbecco’s Modified Eagle’s/F12 (HAM) medium (DMEM/F12) were from Beth Ha-Emek, Biological Industries, Israel.

Sodium azide, trypan blue, p-nitrophenylphosphate, phenylmethylsulfonyl fluoride, leupeptin, benzamidine, aprotinin, DMSO, Tween 20, Tris, 4,6-diamidino-2-phenylindole (DAPI), bovine serum albumin (BSA), Trypsin-EDTA, dihyroethidium (DHE), lipopolysaccharide (LPS), Skim Milk Powder, Poly-L-lysine, horseradish peroxidase (HRP), 1,2-Dioleoyl-sn-glycerol, Triton X-100, β-mercaptoethanol, Percoll, non-essential amino-acids, Diphenyliodonium chloride (DPI) were from Sigma Israel, Rehovot, Israel. Fetal calf serum was from GE Healthcare Life Sciences HyClone Laboratories, Inc., Logan Utah, USA. ECL detection kit for the immunoblot analysis was from PerkinElmer, MA, USA. Pyrrophenone was from Cayman Chemical, Michigan, USA. TNF-α-neutralizing antibody and U0126 (MEK1/2 inhibitor) were from Cell Signaling Technology, Danvers, MA, USA. Interleukin (IL)-4, IL-10, TNF-α, IFN-γ were from PeproTech Asia, NJ, USA.

### Primary microglial cell culture

Microglia were isolated from the brains of mice C57BL 1-day-old pups as previously described [40] with minor modifications. Briefly, the pups were decapitated and the brains were taken out. The tissues were digested by incubation with an enzymatic solution containing papain (116 mM NaCl, 5.4 mM KCl, 26 mM NaHCO3, 1 mM NaH_2_PO_4_, 1.5 mM CaCl_2_, 1 mM MgSO_4_, 0.5 mM EDTA, 25 mM glucose, 1 mM cysteine, and 20 U/ml papain) for 60 min at 37 °C, 5% CO_2_. The enzymatic solution was quenched with 20% FBS in HBSS and centrifuged for 4 min at ×200*g*. A second digestion procedure was performed by treating the brain tissues with 0.5 mg/ml DNase-I (Worthington Biochemical Corp., NJ, USA) for 5 min and gently passing it through a fire-polished Pasteur pipettes several times. Then, the digested tissues were filtered through a 70 micron cell strainer (Corning, NY, USA) and centrifuged at 200*g* for 4 min. The pellet was resuspended in 20% isotonic percoll in HBSS. Fresh HBSS was carefully added and then the tubes were centrifuged at ×200*g* for 20 min with slow acceleration and no brakes. The pellet containing the mixed glial cells were washed with HBSS, centrifuged at ×200*g* for 4 min and then suspended in DMEM-F12 medium (10% FCS, 1% non-essential amino-acids, 11.4 μm β-mercaptoethanol, 10 mM HEPES, 1 mM sodium pyruvate 2 mM L-glutamine, 100 U/ml penicillin, 100 μg/ml streptomycin, and 12.5 U/ml nystatin). The cells were seeded into Poly-L-lysine coated flasks and kept at 37 °C in a humidified atmosphere of 5% CO_2_. The growth medium was replaced with a fresh after 4 days. After two weeks, the microglial cells were separated from the astroglial cell monolayer by shaking the flasks for 1 h at 120 rpm on a rotator shaker and subjected to mild trypsinization with DMEM containing 0.25% Trypsin-EDTA (1:3) for about 90 min at 37 °C and then exchange with fresh DMEM. Then, the isolated microglial cultures were treated with 0.25% Trypsin for approximately 15 min at 37 °C and carefully detached. The cells were suspended with DMEM-F12 (containing 2% FBS 2 mM glutamine, 100 U/ml penicillin, 100 μg/ml streptomycin, and 12.5 U/ml Nystatin) and cultured (6 × 10^5^ cells/ml) in 24 wells on cover-slips coated with Poly-L-lysine at 37 °C in a humidified atmosphere of 5% CO_2_ for a week before the experiment. The purity of microglial cell preparations was confirmed by testing their immunoreactivity to the Iba-1 (Wako Chemicals, Richmond, VA, USA) marker.

### Cell cultures

BV2 immortalized murine microglial cell line was a kind gift from Prof. Rosario Donato (Department of Biochemical Sciences, University of Perugia, Italy). The cells were maintained in DMEM containing 5% FBS 2 mM L-glutamine, 100u/ml penicillin, 100 μg/ml streptomycin, and 12.5 U/ml Nystatin at 37 °C and 5% CO_2_ until they reached confluence. The cells (3.5 × 10^5^ cells/ml) were suspended in DMEM containing 2% FBS, 2 mM L-glutamine, 100 U/ml penicillin, 100 μg/ml streptomycin, and 12.5 U/ml Nystatin and seeded in plates of 24 or 6 wells at 37 °C in a humidified atmosphere of 5% CO_2_.

### Flow cytometry

The microglial cells were suspended in PBS and counted by Trypan Blue. The cells were pre-incubated with rat anti-mouse Fc Blocker (BD Pharmingen, San Jose, CA) at 4 °C for 10 min. For detection of CD40, the cells were incubated with PE anti-mouse CD40 (BioLegend, San Diego, CA) for 2 h on ice in the presence of Fc Blocker. Next, the cells were washed three times with PBS and subjected to fluorescence-activated cell sorter (FACS FC 500, Switzerland, Beckman Coulter) analysis. The median (median of fluorescence intensity) was calculated by subtracting the non-specific fluorescence.

### Immunofluorescence analysis

Microglia were suspended in DMEM (2% FBS, 2 mM L-glutamine, 100 U/ml penicillin, 100 μg/ml streptomycin, and 12.5 U/ml Nystatin) and seeded on cover slips. The cells were fixed with ice-cold methanol for 3 min and then washed with HBSS. For immunofluorescence detection, the fixed microglial cells were incubated with the first antibody 1:50 in 5% BSA/PBS (anti cPLA_2_α (Santa Cruz Biotechnology, CA, USA), anti CD40 (Serotec, Cambridge, UK), anti CD206 (R&D Systems, Minneapolis, USA) Serotec, Oxfordshire, UK) for 90 min at room temperature. The cells were washed three times in HBSS and incubated with Cy3 anti-rabbit, DyLight anti-rabbit, and Cy3 anti-goat (1:50 in 5% BSA/PBS; Jackson ImmunoResearch Laboratories, Inc., PA, USA) for 60 min at room temperature. The cells were washed three times in HBSS, and the nuclei were stained with DAPI. Then, final wash was performed and the cells were taken to fluorescence microscope analysis (Olympus, BX60, Hamburg, Germany).

### Intracellular superoxide anion assay

O_2_^−^ production was measured using dihyroethidium (DHE). The cells were incubated in a 24-well plate on cover slips for 24 h at 37 °C. The next day the medium was replaced with heated HBSS containing 10 μm DHE, and the cells were incubated for 45 min at 37 °C. Then, the cells were stimulated with IFN-γ or LPS for 15 min. Then, the cells were stained with DAPI, washed, and fixed with ice-cold methanol for 3 min. the fluorescence intensity was measured by fluorescence microscope (Olympus, BX60, Hamburg, Germany).

### Inhibition of cPLA_2_α expression using antisense oligonucleotides

An oligodeoxy-nucleotide antisense (tcaaaggtctcattccaca) and its corresponding sense with phosphorothioate modifications on the last three bases at both 5′ and 3′ ends were used as described in our previous article [35]. The specificity to cPLA_2_α was analyzed by blast search program and was demonstrated in our previous study [31].

### Immunoblot analysis

Microglial cell lysates were prepared using lysis buffer containing: 2% Triton X-100, 50 mM HEPES (pH 7.5), 150 mM NaCl, 1 mM EDTA, 1 mM EGTA, 10% glycerol, 10 μm MgCl_2_, 10 μg/ml leupeptin, 1 mM phenylmethylsulphonylfluoride, 10 μg/ml aprotonin, 1 mM benzamidine, 20 mM para-nitrophenyl phosphate, 5 mM sodium orthovanadate, 10 mM sodium fluoride, and 50 mM β-glycerophosphate). Cell lysates were analyzed by SDS-PAGE on 9% gels. The amount of protein in each sample was quantified with the Pierce BCA Proteins Assay using BSA standards. The resolved proteins were transferred to nitrocellulose and blocked in 5% BSA in TBS-T (10 mM Tris, 135 mM NaCl, pH 7.4, 0.1% Tween 20). The blots were incubated overnight at 4 °C with primary antibodies (anti-cPLA_2_α and anti-phospho-(serine-505)-cPLA_2_α from Sigma, anti-NF-κB p65, anti-phospho-(serine-536)-NF-κB p65, anti phopho-p44/42 ERK1/2 (Thr202/Tyr204), anti-p44/42 ERK1/2, anti-STAT1α, anti-phospho-(serine-727)-STAT1α, anti-phospho-(Thr-701)-STAT1α from Cell Signaling, MA, USA; washed and incubated with peroxidase-conjugated secondary antibodies (Amersham Pharmacia Biotech, NJ, USA) for 1.5 h at room temperature. Detection of immunoreactive bands was carried out using enhanced chemiluminescence. Changes in protein expression or phosphorylation were quantified by densitometry using ImageJ program. The intensity of each band was divided by the intensity of each total protein band and expressed as arbitrary units. The quantitative measurements are adequate to determine the changes of each protein in the same immunoblot.

### Separation of plasma membranes and immunoprecipitation

Plasma membranes were separated as described before ([41]). Cell 10^8^/ml suspended in relaxation buffer (100 mM KCl, 3 mM NaCl, 3.5 mM MgCl2, 1.25 mM EGTA, 1 mM ATP, 10 mM PIPES, pH7.4) containing 1 mM PMSF and 100 μm leupeptin at 4 °C and sonicated , resulting in 95% cell breakage. After centrifugation (5 min; ×15,600*g*) to remove the granules, nuclei, and unbroken cells, the supernatant was centrifuge in a Beckman Airfuge (Beckman Instrument, Fulletron, CA) 30 min; ×134,000*g* to obtain cell membrane pellet and cytosol supernatant. The membranes were suspended at 10^9^ cell equivalent/ml in 0.34 sucrose/half-strength relaxation buffer. The microglial cell membranes subjected to immunoprecipitation with goat anti-serum raised against recombinant p47^phox^ (gift from Dr. T Leto, NIAID, NIH, Bethesda, USA). Immunoprecipitation was at a final volume of 0.5 ml at 4 °C. Recombinant protein A–Sepharose beads (Zymed Laboratories Inc., CA, USA) were added to each sample, and the samples were tumbled end-over-end for 1 h. The beads were then washed six times with lysis buffer boiled in lamely buffer and subjected to SDS-PAGE analysis.

*TNF-a detection*–using mouse TNF-α high sensitivity ELISA, eBioscience, Vienna, Austria.

### Statistical analysis

Significant differences between the parameters evaluated were determined by ANOVA using GraphPad Prism 5 (GraphPad Software Inc., San Diego, CA, USA) followed by multiple comparisons Bonferroni post hoc correction. *p* value less than 0.05 were considered statistically significant.

## Results

### cPLA_2_α upregulation regulates the overexpression of CD40 in microglia

**Fig. 1:**
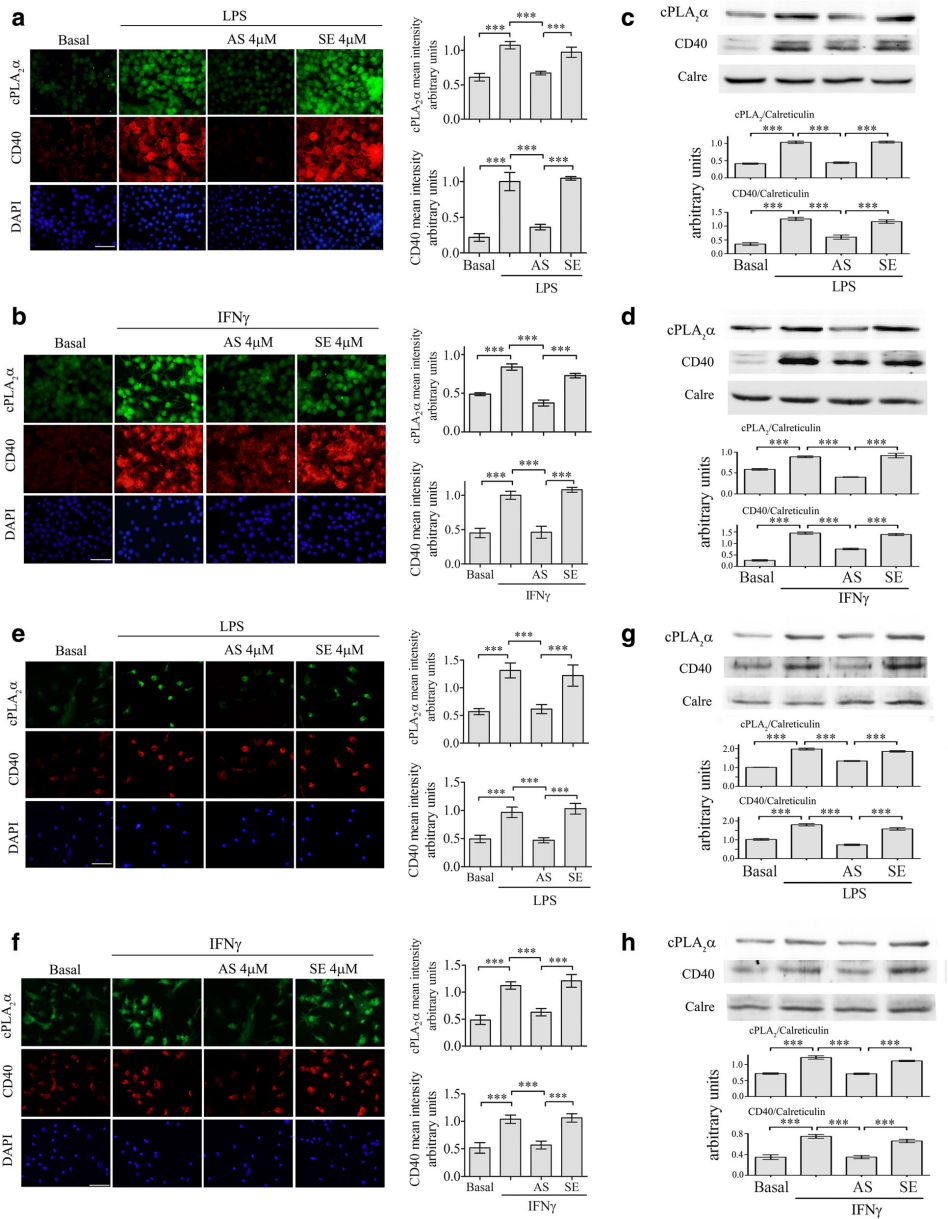
Elevated CD40 expression by LPS or IFNγ in the BV-2 and primary microglia cells is regulated by cPLA_2_α. A representative double-immunofluorescence staining of cPLA_2_α (*green*) and CD40 (*red*) in unstimulated or stimulated microglia by LPS or IFNγ in the absence or presence of AS or sense (SE). DAPI staining shows cell nuclei. The BV-2 cells were treated with (**a**) 50 ng/ml LPS or (**b**) 10 ng/ml IFNγ for 24 h. The mouse primary microglia cells were treated with (**e**) 50 ng/ml LPS or (**f**) 25 ng/ml IFNγ for 48 h. *Scale bars* = 50 μm. Four micrometer AS or the corresponding sense (SE) were added to the cultures 24 h before addition of the stimuli. The intensity of CD40 or cPLA_2_α were quantitated for the cell and expressed in the *bar graph* as arbitrary units. A representaive immunoblot analysis of cPLA_2_α and CD40 for the BV-2 microglia cells (**c**, **d**) and primary mouse microglia (**g**, **h**) treated as (**a**), (**b**), (**e**), and (**f**). The intensity of each cPLA_2_α or CD40 band after quantification by densitometry was divided by the intensity of each calreticulin (Calre) band and expressed as arbitrary units. The *bar graphs* are the mean ± SE from three independent experiments. (***p < 0.0001)

Addition of 50 ng/ml LPS or 10 ng/ml IFNγ to BV-2 microglia cell line for 24 h caused a significant (*p* < 0.0001) elevation of and cPLA_2_α of CD40 protein expression, as shown in the double-immunofluorescence staining analysis (Fig. 1a, b). To determine whether cPLA_2_α upregulation is involved in the induction of CD40 by either LPS or IFNγ, cPLA_2_α upregulation was prevented by a specific antisense oligo-deoxy-nucleotide against cPLA_2_α (AS). As shown in Fig. 1a, b, impeding cPLA_2_α upregulation by addition of 4 μm AS 24 h prior to addition of LPS or of IFNγ prevented CD40 protein induction. Incubation with the corresponding sense that had no effect on the elevation of cPLA_2_α protein expression by either of the inducers did not affect the elevation of CD40 protein expression. To further support these results, the reduction of both cPLA_2_α and CD40 in the presence of AS was validated by western blot analysis (Fig. 1c, d). Similar results were obtained in primary mouse microglia cultures. As shown in the immunofluorescence staining (Fig. 1e, f) and western blot analysis (Fig. 1g, h), preventing cPLA_2_α upregulation prevented the elevated CD40 protein expression induced by LPS or IFNγ. BV-2 is an immortalized mouse microglia cell line that is reported to share many characteristics with primary microglia [42]. Since the BV-2 cells act similarly to primary microglia cultures, they were used to study the role of cPLA_2_α in the regulation of CD40 upregulation by either LPS or IFNγ.

**Fig. 2:**
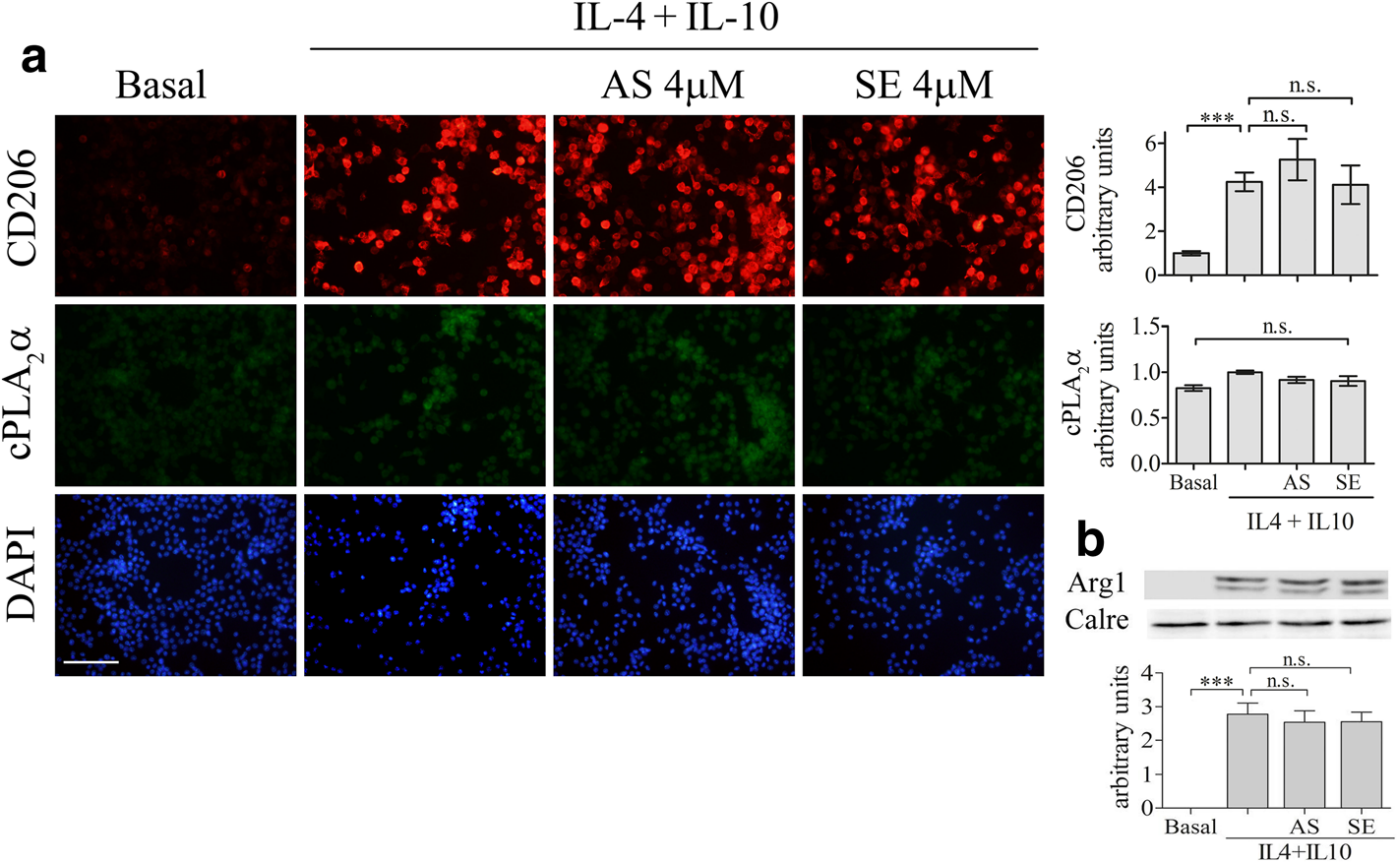
cPLA_2_α is not involved in the induction of the BV-2 cells towards M2 phenotype induced by IL-4 + IL-10. A representative double-immunofluorescence staining of cPLA_2_α (*green*) and CD206 (*red*) in the unstimulated or stimulated BV-2 cells with IL-4 (20 ng/ml) + IL-10 (20 ng/ml) for 24 h in the absence or presence of AS or SE. DAPI staining shows cell nuclei. *Scale bars* = 100 μm. **a** The intensity of CD206 or cPLA_2_α were quantitated and expressed in the *bar graph* as arbitrary units. The *bar graphs* are the mean ± SE from three independent experiments. **b** A representative immunoblot analysis of arginase 1 protein expression in the cells treated as in **a**. The intensity of each Arg1 band after quantification by densitometry was divided by the intensity of each calreticulin band and expressed as arbitrary units. The *bar graphs* are the mean ± SE from three independent experiments. (***p < 0.0001, *n.s*. not significant)

To study whether cPLA_2_α affect microglia induction towards M2 phenotype, the BV-2 cells were cultured with 20 ng/ml IL4 + 20 ng/ml IL10 for 24 h. IL4 + IL10 caused a significant (*p*< 0.0001) elevation of CD206 (Fig. 2a). Pre-incubated of the BV-2 cells with AS or the corresponding sense for 24 h prior to addition of IL4 + IL10 for 24 h did not affect the elevated expression of CD206. Similar results were obtained with respect to the induction of arginase 1, another marker of M2 microglia; the presence of AS or sense did not affect the elevated expression of arginase 1 induced by IL4 + IL10 detected by immunoblot analysis (Fig. 2b).

### The location of cPLA_2_α in the signal transduction leading to CD40 upregulation by LPS

**Fig. 3:**
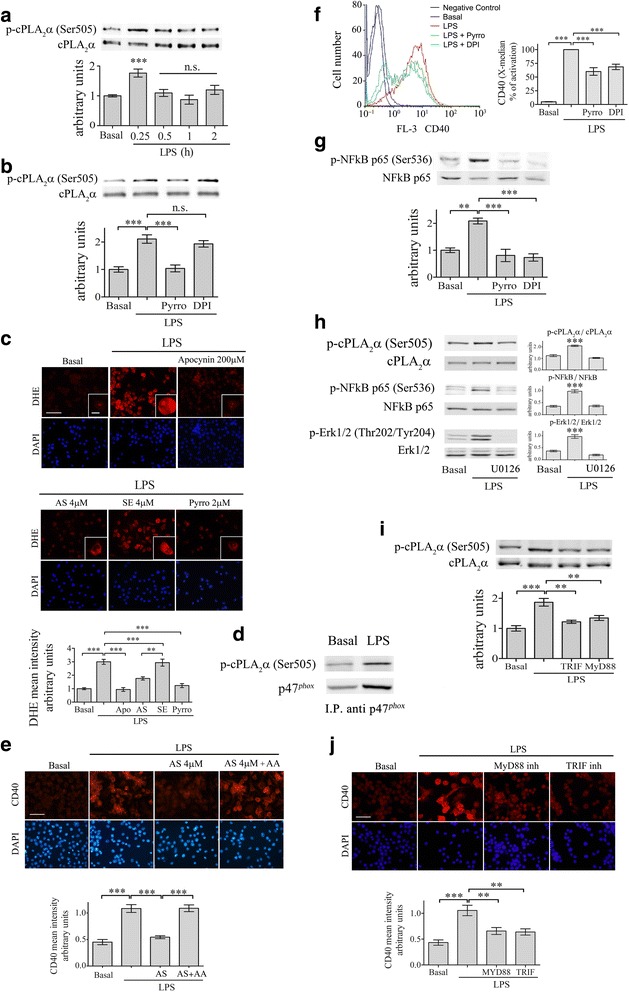
cPLA_2_α activates NFκB through activation of NOX2-NADPH oxidase in the BV-2 microglia cells under LPS stimulation. **a** A representative immunoblot analysis of the kinetics of cPLA_2_α phosphorylation induced by 50 ng/ml LPS, out of three independent experiments. The intensity of each phosphorylated cPLA_2_α (p-cPLA_2_α Ser-505) band after quantification by densitometry was divided by the intensity of each cPLA_2_α band and expressed as arbitrary units. The *bar graphs* are the mean ± SE from three independent experiments. **b** The BV-2 cells were treated with 2 μm pyrrophenone (Pyrro) or 5 μm DPI for 60 min before stimulation with 50 ng/ml LPS for 15 min. The intensity of phosphorylated cPLA_2_α was quantitated by densitometry as described in (**a**). The *bar graphs* are the mean ± SE from three independent experiments. **c** The effect of cPLA_2_α inhibition on superoxide production in the unstimulated or stimulated BV-2 cells with 50 ng/ml LPS for 15 min was detected by DHE reduction. Two micrometer pyropheonoe (Pyrro) or 200 μm apocynin (used as a positive control) were added to the cells 60 min before stimulation with LPS. AS or sense were added 24 h prior to addition of LPS. DAPI staining shows cell nuclei. *Scale bars* large = 50 μm, insert = 20 μm. The intensity of reduced DHE was quantitated and expressed in the *bar graph* as arbitrary units. The *bar graphs* are the mean ± SE from three independent experiments. **d** Immunoprecipitation of p47^phox^ and phoshpo cPLA_2_α (pcPLA_2_α) in the membrane fraction of unstimulated microglia and stimulated with LPS for 15 min. Shown a representative immunoblot of three experiments. **e** Addition of 10 μM arachidonic acid together with LPS to cells pretreated for 24 h with antisense against cPLA_2_α restored the expression of CD40 protein. Shown a representative immunofluorescence staining of CD40. DAPI staining shows cell nuclei. The intensity of CD40 was quantitated for the cell and expressed in the *bar graph* as arbitrary units. *Scale bars* = 50 μm. The *bar graph* is the mean ± SE from three independent experiments. (***p < 0.0001). **f** FACS analysis of CD40 protein expression in the unstimulated or stimulated BV-2 cells with 50 ng/ml LPS for 24 h in the absence or presence of 2 μm pyrrophenone or 5 μm DPI (added to the cells 60 min before stimulation with LPS). The *bar graphs* are the X-median ± SE from five independent experiments. **g** A representative immunoblot analysis of NF-κB p-65 phosphorylation (p-NFκB p-65 Ser-536) in unstimulated or stimulated BV-2 microglia by 50 ng/ml LPS for 15 min in the absence or presence of 2 μm pyrrophenone or 5 μm DPI. The intensity of each phosphorylated NF-κB p-65 band after quantification by densitometry was divided by the intensity of each NFκB p-65 band and expressed as arbitrary units. The *bar graphs* are the mean ± SE from three independent experiments. **h** A representative immunoblot analysis of phoshpo cPLA_2_α and phospho NF-κB p-65 subunit and phosspho ERK1/2 in unstimulated or stimulated BV-2 microglia by 50 ng/ml LPS for 15 min in the absence or presence of 5 μM OU126. The *bar graphs* are the mean ± SE of the intensity of the quantitated phosphorylated forms divided by the non-phophorylated forms of three independent experiments. **i** The involvement of TRIF and MyD88 pathways in activation of cPLA_2_α in the signaling leading to CD40 upregualtion. The BV-2 cells were incubated with TRIF or MyD88 peptide inhibitors for 60 min before stimulation with 50 ng/ml LPS for 15 min. A representative immunoblot analysis of cPLA_2_α activity, out of three independent experiments is presented. The intensity of phosphorylated cPLA_2_α (p-cPLA_2_α Ser-505) was quantitated by densitometry as described in A. **j** Shown is a representative immunofluorescence analysis of CD40 protein expression in the cells treated as in **i**. DAPI staining shows cell nuclei. *Scale bars* = 50 μm. The intensity of cPLA_2_α and of CD40 was quantitated and expressed in the *bar graph* as arbitrary units. The *bar graph* is the mean ± SE from three independent experiments. (***p < 0.0001,**p < 0.001, *n.s.* not significant)

Next, we aimed to determine the location of cPLA_2_α in the suggested signal transduction pathways induced by either LPS or IFNγ [37–39]. We first focused on the signaling induced by LPS and analyzed the time dependent of cPLA_2_α activation determined by the appearance of its phosphorylated form on serine 505 in BV-2 cells lysates. As shown in Fig. 3a, a significant (*p*< 0.0001) transient activation was detected at 15 min after addition of 50 ng/ml LPS to the BV-2 cells. This phosphorylation was prevented in the presence of 2 μm of pyrrophenone added 60 min before activation (Fig. 3b). Since we have previously reported that cPLA_2_α activity regulates NOX2-NADPH oxidase activity in the phagocytic cells including primary rat microglia [40], we studied whether under LPS stimulation the oxidase is also regulated by cPLA_2_α. Addition of the oxidase inhibitor, 5 μm DPI, prior to LPS added for 15 min did not affect the activation of cPLA_2_α (Fig. 3b) while addition of 4 μm AS for 24 h or pyrrophenone for 60 min prior to addition of LPS for 15 min caused a significant (*p* < 0.0001) inhibition of superoxide production as measured by DHE reduction, similar to that caused by the presence of a specific inhibitor of the oxidase, 200 μm apocynin (Fig. 3c). Pre-incubation of the BV-2 cells with 4 μm of the corresponding sense had no effect on superoxide production. These results indicate that cPLA_2_α activity regulates NOX2-NADPH oxidase activity in BV-2 microglia stimulated by LPS. The immunoprecipitation of the cytosolic subunit of NOX2-NADPH oxidase with the phosphorylated form of cPLA_2_α in the membrane fraction of the activated microglia cells further support the role of cPLA_2_α in the regulation of the oxidase (3D). Our previous studies demonstrated that arachidonic acid restored the inhibited NOX2-NADPH oxidase activity in the absence of cPLA_2_α activity [43]. In line with our previous results, addition of arachidonic acid to the stimulated cells in the presence of antisense against cPLA_2_α restored the expression of CD40 (Fig. 3e). To further support that the NOX2-NADPH oxidase is located in the signal events leading to CD40 upregulation by LPS, the effect of its inhibition was studied on CD40 expression. The presence of a specific inhibitor of NOX2-NADPH oxidase activity (200 μm apocynin) prevented the elevation of CD40 protein expression induced by LPS, as determined by FACS analysis, (Fig. 3f) similar to the effect caused by the presence of the inhibitor of cPLA_2_α activity, pyrrophenone. The results indicate that the NOX2-NADPH oxidase activity participates in the signal transduction pathway leading to CD40 induction by LPS and is located downstream to cPLA_2_α. As shown in Fig. 3g, addition of LPS for 15 min caused activation of NFκB, detected by the phosphorylation of its p65 subunit on serine 536. This phosphorylation was similarly reduced by inhibition of cPLA_2_α or NOX2-NADPH oxidase activity by the presence of either pyrrophenone or DPI, respectively. The presence of a specific inhibitor of MEK1/2 activation, U0126, caused inhibition of ERK1/2, cPLA_2_α, and NFκB activation induced by LPS as detected by their phosphorylated forms (Fig. 3h). Taken together, these results suggest that early after the addition of LPS to the BV-2 cells, ERK activates cPLA_2_α that in turn activates the assembled NOX2-NADPH oxidase that mediates the activation of NF-κB.

Next, we determined whether MyD88- or TRIF- pathways in the signal events induced by LPS are capable of activating cPLA_2_α. We used specific peptide inhibitors for either MyD88 signaling by inhibiting its homodimerization or TRIF signaling by interfering with TLR-TRIF interaction. As shown in the immunoblot, each peptide inhibitor inhibited the activation of cPLA_2_α detected by its phosphorylated form induced by LPS, to the levels detected in the unstimulated cells (Fig. 3i). Moreover, either of the inhibitors totally prevented the induction of CD40 induced by LPS, suggesting that both pathways participate in cPLA_2_α activation and in CD40 upregulation (Fig. 3j).

**Fig. 4:**
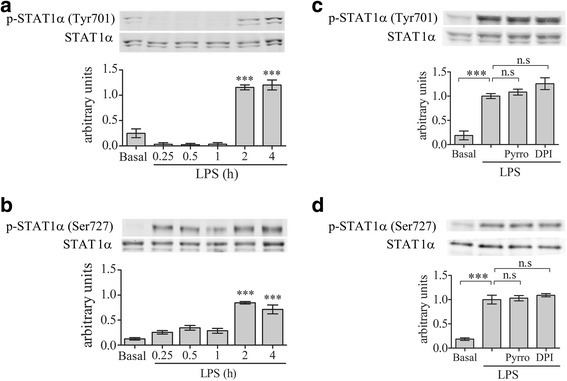
STAT1α activation is not under regulation of cPLA_2_α or NOX2-NADPH oxidase in the BV-2 microglia cells under LPS stimulation. A representative immunoblot analysis of the kinetics of STAT1α phosphorylation on (**a**) tyrosine 701 or (**b**) serine 727 induced with 50 ng/ml LPS. A representative immunoblot analysis of STAT1α phosphorylation on (**c**) tyrosine 701 or on (**d**) serine 727 in unstimulated or stimulated with 50 ng/ml LPS for 4 h in the absence or presence of 2 μm pyropheonoe (Pyrro) or 5 μm DPI added to the cells 1 h before stimulation. The intensity of each phosphorylated STAT1α (p-STAT1α) band after quantification by densitometry was divided by the intensity of each STAT1α band and expressed as arbitrary units. The *Bar graphs* are the mean ± SE from three experiments (***p < 0.0001, *n.s*. not significant)

Since activation of STAT1α was reported to signal the induction of CD40 by LPS [39], we studied whether STAT1α activation is dependent on cPLA_2_α activity. Kinetics analysis of STAT1α activation detected by its phosphorylation revealed that significant phosphorylation of STAT1α on tyrosine 701 (Fig. 4a) and on serine 727 (Fig. 4b) was detected at 4 h of stimulation with LPS. Neither tyrosine 701 nor serine 727 phosphorylation induced by LPS for 4 h were affected by the presence of cPLA_2_α inhibitor, pyrrophenone, or the presence of the NOX2-NADPH oxidase inhibitor, DPI (Fig. 4c, d), suggesting that STAT1α is not under cPLA_2_α regulation in the signal cascade leading to CD40 induction by LPS in the BV-2 cells.

### The location of cPLA_2_α in the signal transduction leading to CD40 upregulation by IFNγ

**Fig. 5:**
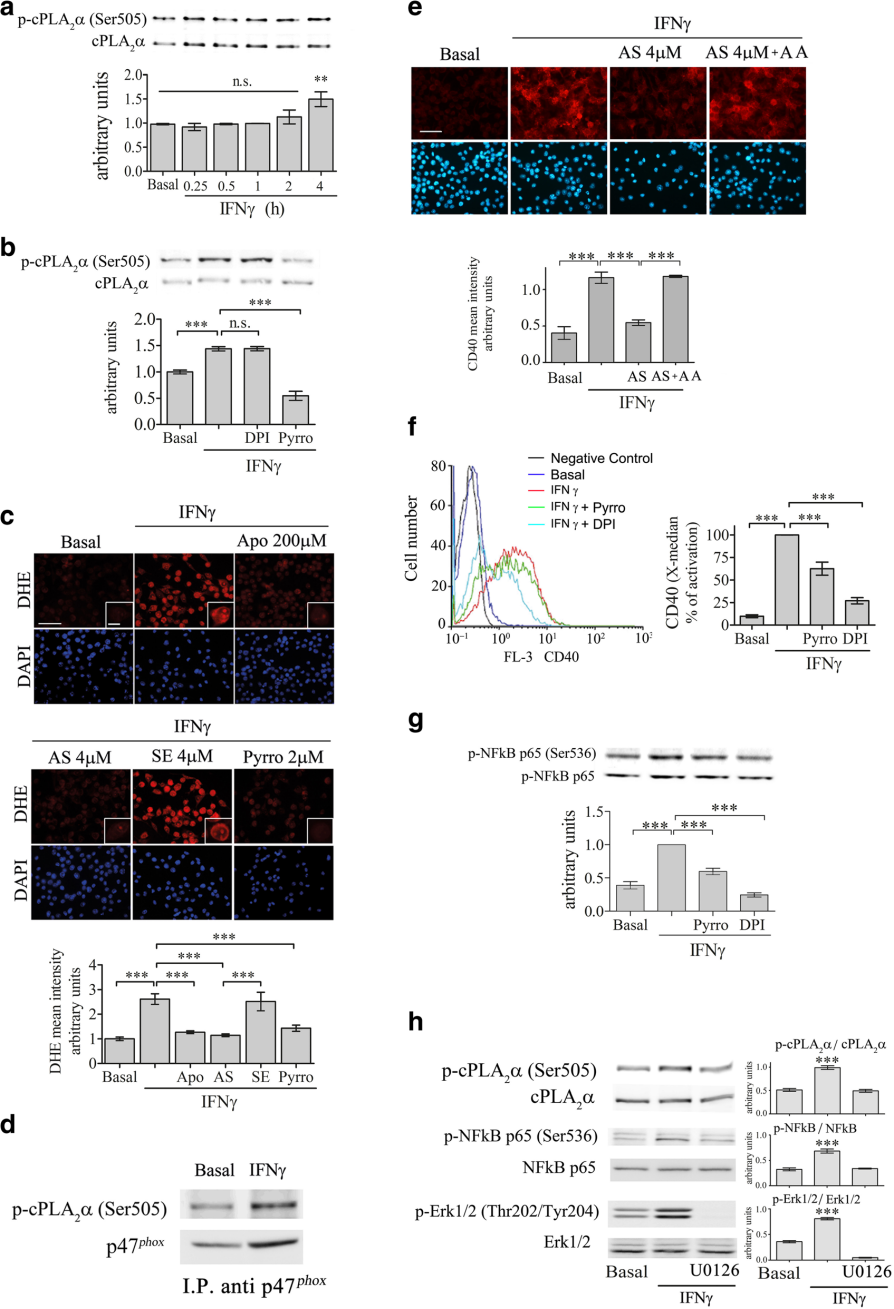
cPLA_2_α activates NF-κB through activation of NOX2-NADPH oxidase in the BV-2 microglia cells under IFNγ stimulation. **a** A representative immunoblot analysis of the kinetics of cPLA_2_α phosphorylation induced by 10 ng/ml IFNγ, out of three independent experiments. The intensity of phosphorylated cPLA_2_α (p-cPLA_2_α Ser-505) was quantitated by densitometry as described in Fig. 3a. **b** The BV-2 microglia cells were treated with 2 μm pyrrophenone (Pyrro) or 5 μm DPI for 1 h before stimulation with 10 ng/ml IFNγ for 4 h. Phosphorylated cPLA_2_α (p-cPLA_2_α Ser-505) intensity was quantitated by densitometry as described in Fig. 3a. The *bar graphs* are the mean ± SE from three independent experiments. **c** The effect of cPLA_2_α inhibition on superoxide production in the unstimulated or stimulated BV-2 cells with 10 ng/ml IFNγ for 4 h detected by DHE reduction. Two micrometer pyropheonoe (Pyrro) or 200 μm apocynin (used as a positive control) were added to the cells 60 min before stimulation with IFNγ for 4 h. AS or sense (SE) were added 24 h prior to addition of IFNγ. DAPI staining shows cell nuclei. The intensity of reduced DHE was quantitated and expressed in the *bar graph* as arbitrary units. *Scale bars* large = 50 μm, insert = 20 μm. The *bar graphs* are the mean ± SE from three independent experiments. **d** Immunoprecipitation of p47^phox^ and phoshpo cPLA_2_α (pcPLA_2_α) in the membrane fraction of unstimulated microglia and stimulated with IFNγ for 4 h. Shown a representative immunoblot of three experiments. **e** Addition of 10 μM arachidonic acid togeher with IFNγ to the cells pre-treated for 24 h with antisense against cPLA_2_α restored the expression of CD40 protein. Shown a representative immunofluorescence staining of CD40. DAPI staining shows cell nuclei. The intensity of CD40 was quantitated for the cell and expressed in the *bar graph* as arbitrary units. *Scale bars* = 50 μm. **f** FACS analysis of CD40 protein expression in the unstimulated or stimulated BV-2 cells with10 ng/ml IFNγ for 24 h in the absence or presence of 2 μm pyrrophenone (Pyrro) or 5 μm DPI added to the cells 1 h before stimulation. The *bar graphs* are the X-median ± SE from five independent experiments. **g** A representative immunoblot analysis of phosphorylated NF-κB p65 (p-NFκB p-p65(Ser-536) in unstimulated or stimulated BV-2 microglia by 10 ng/ml IFNγ for 4 h in the absence or presence of 2 μm pyrrophenone (Pyrro) or 5 μm DPI. The intensity of each p-NFκB p-p65(Ser-536) band after quantification as described in Fig. 3g. The *bar graphs* are the mean ± SE from three independent experiments. **h** A representative immunoblot analysis of phoshpo cPLA_2_α and phospho NF-κB p-65 subunit and phosspho ERK1/2 in unstimulated or stimulated BV-2 microglia by 10 ng/ml IFNγ for 4 h in the absence or presence of 5 μM OU126. The *bar graphs* are the mean ± SE of the intensity of the quantitated phosphorylated forms divided by the non-phophorylated forms of three independent experiments. (***p < 0.0001)

To determine the location of cPLA_2_α in the signal transduction events leading to CD40 protein induction by IFNγ, we first studied the time-dependent activation of cPLA_2_α. As shown in Fig. 5a, a significant (*p* < 0.0001) cPLA_2_α activation detected by its phosphorylation on serine 505 appeared at 240 min of stimulation with 10 ng/ml IFNγ in BV-2 cell lysates. This phosphorylation was prevented in the presence of 2 μm pyrrophenone but not by the presence of 5 μm DPI (Fig. 5b), suggesting that the NOX2-NADPH oxidase is downstream to cPLA_2_α. Inhibition of cPLA_2_α activity by addition of either AS for 24 h or pyrrophenone for 60 min prior to stimulation by IFNγ for 4 h caused inhibition of NOX2-NADPH oxidase activity detected by DHE reduction that was similar to that achieved in the presence of 200 μm apocynin (Fig. 5c), while the presence of sense had no effect. The immunoprecipitation of NOX2-NADPH oxidase cytosolic subunit p47^phox^ with phopho-cPLA_2_α in the membrane fraction of the activated mircroglia cells at 4 h further support the role of cPLA_2_α in regulating the oxidase activity (Fig. 5d). Addition of arachidonic acid to the IFNγ stimulated cells in the presence of antisense against cPLA_2_α restored the expression of CD40 (Fig. 5e). The presence of DPI or pyrrophenone significantly inhibited the induction of CD40 as shown by FACS analysis (Fig. 5f). These results suggest that NOX2-NADPH oxidase is downstream to cPLA_2_α in the signal transduction pathway leading to CD40 upregulation induced by IFNγ. Inhibition of cPLA_2_α or NOX2-NADPH oxidase by the presence of either pyrrophenone or DPI inhibited NF-κB activity detected by the phosphorylation of its p65 subunit on serine 536 at 4 h of stimulation with IFNγ (Fig. 5g), suggesting that NF-κB is downstream to cPLA_2_α and NOX2-NADPH oxidase. The presence of a specific inhibitor of MEK1/2 activation, U0126, caused inhibition of ERK1/2, cPLA_2_α, and NF-κB activation at 4 h of induction by IFNγ as detected by their phosphorylated forms (Fig. 5h).

**Fig. 6:**
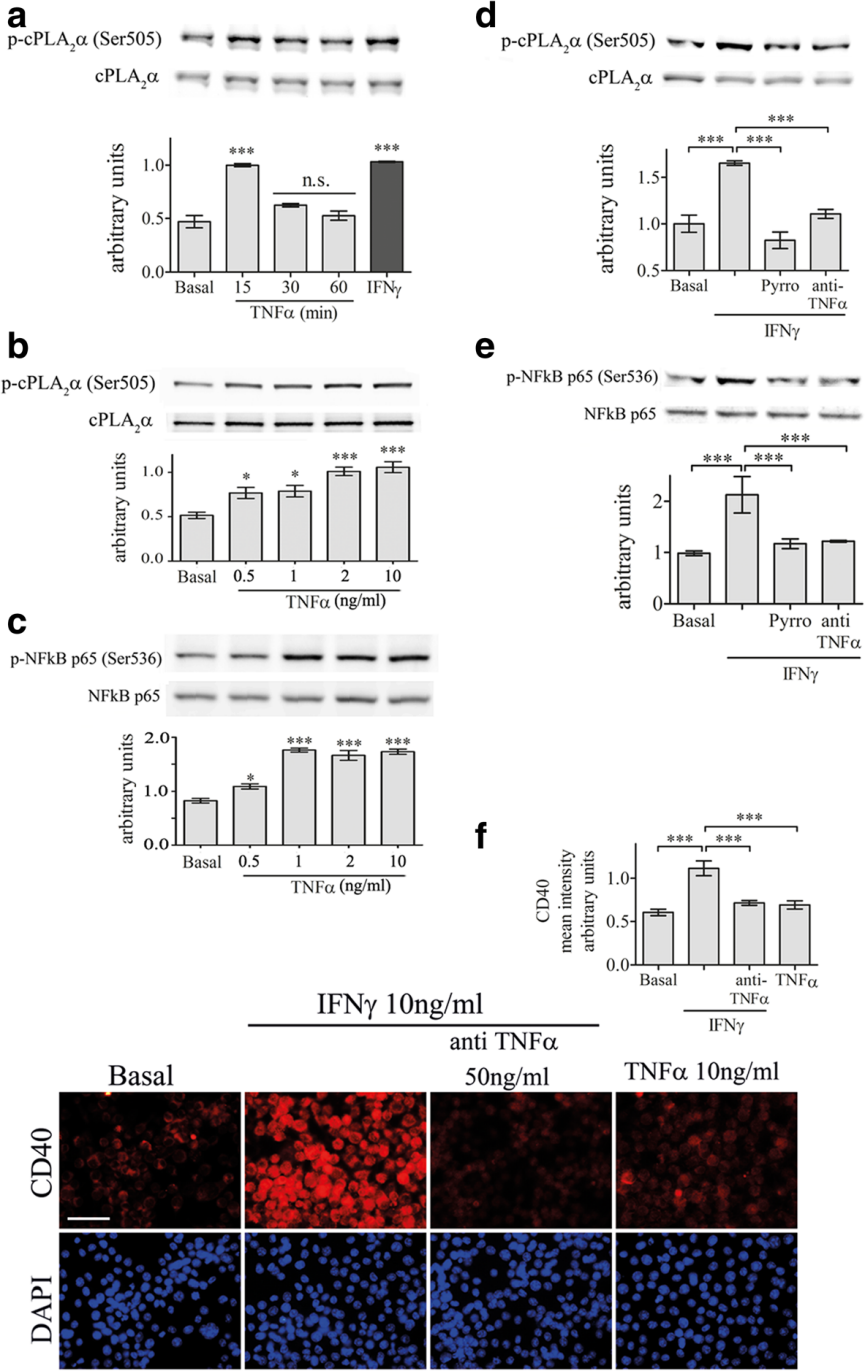
Endogenously produced TNF-α regulates cPLA_2_α activation in the BV2 cells under IFNγ stimulation. **a** A representative immunoblot analysis of the kinetics of cPLA_2_α activation detected by its phosphorylated form induced by 10 ng/ml TNF-α in BV-2 microglia lysates. A representative immunoblot analysis of a dose-dependent activation of the cPLA_2_α (**b**) and of NF-kB (**c**) detected by their phosphorylated induced by TNF-α in BV-2 microglia lysates. Representative immunoblot analysis of (**d**) cPLA_2_α phosphorylation on Ser-505 and (**e**) NF-kB p-65 phosphorylation on Ser-536 in unstimulated or stimulated BV-2 microglia by 10 ng/ml IFNγ for 4 h in the absence or presence of 2 μm pyrrophenone (Pyrro) or 50 ng/ml TNF-α-neutralizing antibody (anti-TNF-α) added to the cells 60 min before stimulation with IFNγ stimulation. The intensity of each p-cPLA_2_α(Ser-505) or p-NFκB p65(Ser-536) band after quantification by densitometry was divided by the intensity of each cPLA_2_α or NFκB p65 band, respectively, and expressed as arbitrary units. The results are the mean ± SE from four experiments. **f** Immunofluorescence analysis of CD40 protein expression in unstimulated or stimulated BV-2 microglia with 10 ng/ml IFNγ for 24 h in the absence or presence of 50 ng/ml TNF-α-neutralizing antibody or stimulated by 10 ng/ml TNF-α. *Scale bars* = 50 μm. The intensity of CD40 was quantitated and expressed in the *bar graph* as arbitrary units. The *bar graph* is the mean ± SE from four independent experiments. DAPI staining shows cell nuclei. (***p < 0.0001, *n.s.* not significant)

It was reported that TNF-α is secreted from macrophages and microglia after 4 h of stimulation [37] and has an autocrine effect on the cells. To determine whether the activation cPLA_2_α is mediated by endogenous release of TNF-α, we first studied the time-dependent activation of cPLA_2_α by TNF-α. As shown in Fig. 6a, TNF-α caused a rapid and transient activation of cPLA_2_α that was similar to that induced by IFNγ for 4 h. We then measured the release of TNF-α form microglia stimulated by IFNγ (Table 1). TNF-α could be significantly detected in the supernatant of microglia cultures for 4 h with IFNγ. The dose-dependent activation of cPLA_2_α by TNF-α showed that cPLA_2_α was significantly (*p* < 0.01) activated by 0.5 ng/ml TNF-α, while 2 and 10 ng/ml were yet more significant (*p* < 0.0001) with a similar effect (Fig. 6b). A similar dose-dependent effect was detected for NF-kB activated by TNF-α (Fig. 6c). To determine the role of the released TNF-α in activation of cPLA_2_α and of NF-κB by IFNγ, its autocrine effect was prevented by pre-incubation of the cells with anti-TNF-α-neutralizing antibody before stimulation. The presence of anti-TNF-α prevented the activation of cPLA_2_α as detected by its phosphorylated forms on serine 505 (Fig. 6d) and prevented the activation of NF-κB p-65 as detected by it phosphorylation on serine 536 (Fig. 6e) at 4 h of IFNγ stimulation. As shown in Fig. 6f, the presence of anti-TNF-α prevented the induction of CD40 by IFNγ in the BV-2 microglia cells. Addition of TNF-α alone to the cells did not induce CD40 protein expression, as expected since both NF-kB and STAT1 transcription factors are required for the induction of CD40 by IFNγ, while TNF-α was shown to activate only NF-κB that is located downstream to STAT1α in the signal events [38].

**Table 1:** TNF-α secretion induced by INFγ

Medium treated	IFNγ treated (4 h)
0.06 ± 0.004	2.7 ± 0.05
Cells were treated with 10 ng/ml IFNγ	

The results are means ± SED from three independent experiments

There is a significant difference between the treated and non-treated cells (*p* < 0.0001)

**Fig. 7:**
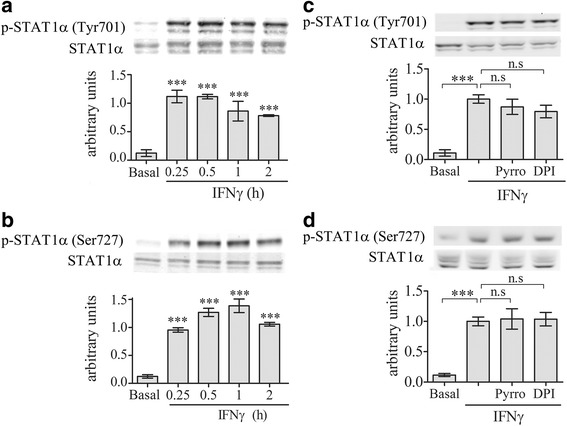
cPLA_2_α or NADPH oxidase did not affect STAT1α activation under IFNγ stimulation. A representative immunoblot analysis of the kinetics of STAT1α phosphorylation on (**a**) tyrosine 701 or (**b**) serine 727 induced by 10 ng/ml IFNγ. A representative immunoblot analysis of STAT1α phosphorylation on (**c**) tyrosine 701 or on (**d**) serine 727 in unstimulated or stimulated BV-2 microglia by 10 ng/ml IFNγ for 15 min in the absence or presence of 2 μm pyrrophenone (Pyrro) or 5 μm DPI (added to the cells 60 min before stimulation). The intensity of each phosphorylated STAT1α (p-STAT1α) band was quantitated as described in Fig. 4. The *Bar graphs* are the mean ± SE from three experiments (***p < 0.0001, *n.s.* not significant)

Addition of IFNγ caused a rapid and significant (*p* < 0.0001) activation of STAT1α on either serine 727 or tyrosine 701, detected at 15 min of activation (Fig. 7a, b). Both phosphorylation were not affected by the presence of cPLA_2_α inhibitor, pyrrophenone, or the presence of NOX2-NADPH oxidase inhibitor, DPI (Fig. 7c, d), suggesting that STAT1α is not under cPLA_2_α regulation in the signal cascade leading to CD40 induction by IFNγ in the BV-2 cells.

## Discussion

The present study shows that cPLA_2_α is involved in the induction of CD40 by either LPS or IFNγ. Reduction of cPLA_2_α upregulation by a specific antisense or inhibition of cPLA_2_α activity by a specific inhibitor prevented the induction of CD40 protein expression by either LPS or IFNγ. The results suggest that cPLA_2_α has a direct role in CD40 upregulation, a feature of the pro-inflammatory M1-phenotype. In accordance with this view, the regulatory role of cPLA_2_α in the induction of several characters of M1 phenotype in microglia and macrophages, such as iNOS, COX2, NOX2-NADPH oxidase as well as production of eicosanoids and pro-inflammatory mediators, was reported by us and others [40, 44]. cPLA_2_α, however, is not involved in the transformation to M2-phenotype, as its protein level was not elevated by addition of IL4 + IL10, and the presence of AS did not affect the significant induction of CD206 or of arginase 1 in microglia. In accordance with our results, it was reported that the antiinflammatory cytokines IL4 or IL10 by themselves did not affect cPLA_2_α activation or biosynthesis [45, 46], further supporting the role of cPLA_2_α in inflammatory processes.

The results of the present study show that superoxides generated by NOX2-NADPH oxidase participate in upregulation of CD40 expression induced by LPS or IFNγ in microglia, since inhibition of NOX-2 NADPH oxidase prevented the induction of CD40. We show here that in BV-2 microglia cell line, inhibition of the activation of cPLA_2_α induced by either LPS or IFNγ, as demonstrated by the use of antisense against cPLA_2_α or the specific inhibitor of cPLA_2_α activity, pyrrophenone, inhibited the production of superoxides by the NOX2-NADPH oxidase. Inhibition of the oxidase activity did not affect cPLA_2_α activation detected by its phosphorylated form. These results suggest that the NOX2-NADPH oxidase is regulated by cPLA_2_α in microglia stimulated with either LPS or IFNγ, that is similar to ours and other studies related to the various phagocytic cells stimulated with a variety of agonists [31, 40, 44, 47–50]. We show here that phoshpo-cPLA_2_α translocated to the cell membranes of activated microglia, where it binds p47^phox^ subunit of NOX2-NADPH oxidase, in accordance with our previous studies in other phagocytic cells as well as in primary rat microglia [40, 43, 48, 50]. The binding between p-cPLA_2_α and 47^phox^ was detected at 15 min when the microglia cells were stimulated with LPS and at 4 h when stimulated with IFNγ in correlation with the detection of superoxide production and the kinetic of cPLA_2_α phosphorylation by the two stimuli. Our previous study [43] demonstrated that arachidonic acid activated the assembled oxidase in activated cPLA_2_α-deficient cells, although the precise mechanism is not known. The restoration of CD40 upregulation in the activated cells that were pretreated with AS against cPLA_2_α by addition of arachidonic acid is probably due to the activation of the NOX2-NADPH oxidase. The activation of cPLA_2_α at 15 min by LPS and at 4 h by IFNγ was mediated by ERK activation since the presence of MEK inhibitor inhibited cPLA_2_α activation in accordance with ours and other earlier studies [44, 51].

The involvement of two transcription factors, NF-κB and STAT1α, was reported in the signal transduction pathways leading to induction of CD40 by either LPS or IFNγ [37–39]. While NF-κB was shown to be rapidly activated by LPS, it was activated only at 4 h following exposure to IFNγ. In contrast, STAT1α was rapidly activated by IFNγ and only at 4 h by LPS. Time-dependent activation of cPLA_2_α detected by its phosphorylation on serine 505 revealed that cPLA_2_α is rapidly activated by LPS and only considerably later (4 h) by IFNγ, that is in accordance with a previous report [44]. We show in the present study that the kinetic of activation of cPLA_2_α coincided with the kinetic of NF-kB activation and that the activation of cPLA_2_α is required for the activation of NF-κB in BV-2 microglia cell line, a finding consonant with our earlier study in microglia activated with amyloid beta [40]. While superoxide production by NOX2-NADPH oxidase is extremely important for killing invading pathogens, it is also an important activator of diverse cell signaling pathways such as mitogen activated protein kinase and NF-κB to regulate the expression of genes encoding a variety of pro-inflammatory factors [40, 52, 53]. The activation of NF-κB by either LPS or IFNγ shown in the present study detected by the phosphorylation of its p-65 subunit on serine 536 is probably mediated by superoxides produced by the NOX2-NADPH oxidase since the inhibition of the oxidase activity prevented NF-kB action. In line with this suggestion, the phosphorylation of p65 NF-kB RelA on Ser-536 is known to be redox-sensitive [54]. The activation of NF-kB by NOX2-NADPH oxidase activity is consistent with our previous studies in microglia and macrophages [40, 55] and with other in various systems and by various agonist [56, 57].

It was reported that the activation of NF-kB under IFNγ stimulation is mediated by an autocrine effect of released TNF-α from the stimulated cells [37]. Consistent with this observation, we show here that the activation of cPLA_2_α and of NF-kB and the induction of CD40 by IFNγ are mediated by an autocrine effect of TNF-α, since TNF-α secretion from the activated cells was detected and the levels of secreted TNF-α activated cPLA_2_α and NF-kB. In addition, the presence of antibodies against TNF-α in microglia stimulated with IFNγ of all three processes were inhibited, suggesting that cPLA_2_α activation by TNF-α regulates the induction of CD40 via NF-kB activation. The activation of cPLA_2_α by TNF-α coincided with other reports in microglia and macrophages [46, 58]. However, addition TNF-α is not sufficient to induce CD40, although it activates cPLA_2_α, probably since it stimulates the activation of NF-κB but not the activation of STAT1α that is also required for CD40 induction.

The activation of cPLA_2_α and NF-κB in the signals leading to CD40 upregulation by LPS is mediated by both MyD88 and TRIF pathways, since inhibition of each pathway inhibited cPLA_2_α and NF-κB activation and abolished CD40 induction. In accordance with our results, the activation of cPLA_2_α by MyD88 and by TRIFF adaptive protein was shown in macrophages stimulated by LPS [59]. The activation of NF-κB leading to CD40 upregulation by LPS was suggested to be mediated only by MyD88 adaptive protein in macrophages [39]. However, several studies reported, similar to our results, that both pathways are mediating NF-κB by TLR4 receptor in macrophages and other cell types [59–61].

## Conclusions

**Fig. 8:**
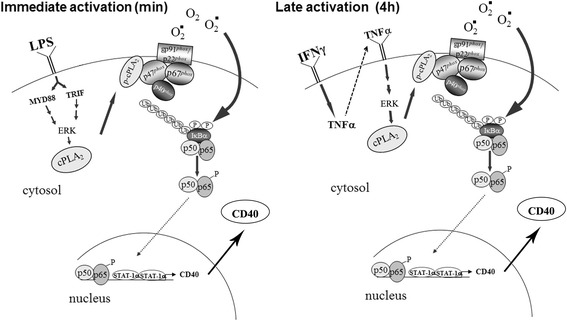
The involvement of cPLA_2_α in the proposed schematic NF-κB signaling pathways of CD40 protein expression induced by LPS or IFNγ (described in the “Discussion”section)

Our results show for the first time that cPLA_2_α regulates CD40 protein induction in microglia by either LPS or IFNγ, and this regulation is mediated via activation of NOX2-NADPH oxidase and NF-κB. STAT1α transcription factor, that was reported to participate in CD40 induction, was early activated by IFNγ and late activated by LPS as detected by the phosphorylation on either serine 727 or tyrosine 701, but this activation was not under cPLA_2_α regulation. As shown in Fig. 8, cPLA_2_α is located in the early event induced by LPS and its activation is mediated by both adaptor proteins, TRIF and MyD88. While, under IFNγ stimulation, cPLA_2_α is activated at a later time (4 h) by the autocrine effect of released TNF-α. Under both stimuli, cPLA_2_α activation is mediated by ERK activity. The role of cPLA_2_α in the induction of the CD40 suggests that cPLA_2_α may serve as an amplifier of the inflammatory response in the CNS and the reduction of its levels in the inflamed organ can lead to therapeutic effect.

## Abbreviations

AS: Antisense oligonucleotide against cPLA_2_α
COX-2: Cyclooxygenase-2
cPLA_2_α: Cytosolic phospholipase A_2_ alpha
DHE: Dihyroethidium
IFNγ: Interferon gamma
iNOS: Inducible nitric oxide synthase
LPS: Lipopolysaccharide
LPS: Lipopolysaccharide
NF-kB: Nuclear factor-kappaB
